# Stage-specific autophagy dynamics in reproductive processes and associated disorders

**DOI:** 10.3389/fcell.2025.1639691

**Published:** 2025-07-28

**Authors:** Jiayi Wang, Shuo Tu

**Affiliations:** ^1^Queen Mary School, Jiangxi Medical College, Nanchang University, Nanchang, China; ^2^School of Basic Medicine, Jiangxi Medical College, Nanchang University, Nanchang, China

**Keywords:** autophagy, gametogenesis and embryonic development, decidualization, spontaneous abortion, preeclampsia

## Abstract

Autophagy is a highly conserved eukaryotic process that degrades cytoplasmic material within lysosomes, and plays a crucial role in cellular development, function and homeostasis. Recent studies have increasingly revealed the connection between autophagy, reproduction, and related disorders. In this review, we summarize the regulatory mechanisms of autophagy and outline recent advances in understanding its role in pregnancy establishment and maintenance, including gametogenesis and embryonic development, decidualization, placentation, and immune regulation advances in understanding its role. Additionally, we discuss potential mechanisms by which altered autophagy contributes to pregnancy complications and reproductive disorders, such as spontaneous abortion, preeclampsia, preterm birth, polycystic ovary syndrome, and endometriosis.

## Introduction

Autophagy is a highly conserved recycling process and a major cellular pathway responsible for the lysosomal degradation of cytoplasmic organelles and proteins. This process plays a vital role in cellular development, function and homeostasis (Clarke and Simon, 2019). Several human disorders are associated with mutations in autophagy related genes, emphasizing that defects in autophagy may contribute to the pathogenesis of human disorders (Mizushima and Levine, 2020). In this review, we discuss the role of autophagy in reproduction and explore the association between autophagy abnormalities and pregnancy-related complications.

Macroautophagy, microautophagy, and chaperone-mediated autophagy (CMA) are the three major forms of autophagy described to date. Macroautophagy (hereafter referred to as autophagy) involves the sequestration of cellular components into double-membrane vesicles called autophagosomes. These autophagosomes subsequently fuse with intracellular lysosomes to form autophagolysosomes, where the sequestered material is degraded (Wen et al., 2022). Although, macroautophagy was initially considered a non-selective process, it has since been shown to selectively degrade specific substances, such as intracellular microbes, damaged mitochondria, and ruptured lysosomes (Mizushima and Levine, 2020; Levine and Kroemer, 2019; Gatica et al., 2018; Pohl and Dikic, 2019). Autophagosomal cargo can be sequestered either non-selectively (bulk autophagy) or through a highly regulated process (selective autophagy) depending on the inducing factors involved (Dikic and Elazar, 2018). Microautophagy can directly engulf cytoplasmic contents or KFERQ-flagged proteins, through endosomal or lysosomal membranous invaginations (Schuck, 2020). Microautophagy performs diverse functions, including metabolic adaptation, biosynthetic transport, and organelle remodeling. In CMA, the heat-shock cognate protein HSPA8/HSC70 recognizes a pentapeptide motif (KFERQ-like) within the substrate protein. The substrate and chaperone complex binds to LAMP2A (lysosomal-associated membrane protein 2A) on the lysosomal membrane, facilitating internalization and subsequent degradation within the lysosome (Bourdenx et al., 2021).

In this review, we specifically focus on macroautophagy. We first describe autophagy regulatory mechanisms and then explore its roles in reproduction, including gametogenesis, embryonic development, decidualization, placentation, and immune regulation at the maternal-fetal interface. Furthermore, we discuss the association between autophagy dysregulation and reproductive-related disorders.

### Regulatory mechanism of autophagy

**FIGURE 1:**
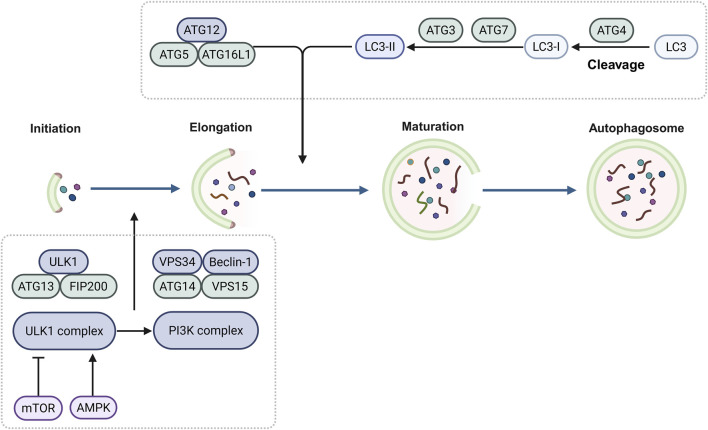
Regulatory mechanisms of autophagy Autophagosome formation is initiated by the ULK complex (ULK1/2, ATG13, ATG101, FIP200), which integrates nutrient/stress signals primarily via mTORC1 inhibition and AMPK activation. The activated ULK complex phosphorylates the PI3K complex (VPS34, Beclin-1, ATG14, VPS15), generating PI3P on phagophore membranes. PI3P recruits conjugation machinery (ATG3, ATG7, ATG5-ATG12-ATG16L1), facilitating LC3/GABARAP lipidation with phosphatidylethanolamine. This lipid-conjugated LC3-II anchors to the expanding autophagosomal membrane, driving its maturation and serving as a key autophagy marker.

During autophagy, several dynamic membrane events contribute to the sequestration of cytoplasmic components within autophagosomes. These events include the appearance, expansion, and closure of phagophores, as well as the maturation, and trafficking, and fusion of the autophagosomes (Zhu et al., 2022). Autophagy is regulated by multiple complexes encoded by evolutionarily conserved autophagy-related (ATG) genes. The products of these ATG genes regulate the autophagosomes formation. Autophagosomes encapsulate the cellular cargo and subsequently fuse with the lysosomes, leading to the degradation of their contents (Debnath et al., 2023; Nishimura and Tooze, 2020). In yeast, more than 40 ATG genes have been identified, among which 15 are considered core ATG genes (*ATG1* to *ATG10*, *ATG12*, *ATG13*, *ATG14*, *ATG16*, and ATG18), essential for both non-selective and selective autophagy (Nishimura and Tooze, 2020). The identification and characterization of the ATG proteins and other autophagy related factors continue to enhance our understanding of the molecular mechanisms regulating autophagy (Figure 1).

Autophagosome formation is initiated by the UNC-51-like kinase (ULK) complex, which includes ULK1 or ULK2, ATG11 (also known as RB1CC1 or FIP200), ATG13 and ATG101. This complex integrates various nutrient and stress signals are received by the ULK complex, with mechanistic target of rapamycin complex 1 (mTORC1) acting as a primary regulator. Under starvation, AMP-activated protein kinase (AMPK) activates ULK1 by phosphorylating Ser 317 and Ser 777, thereby promoting autophagy. During nutrient-rich conditions, elevated mTOR activity inhibits ULK1 activation by phosphorylating ULK1 Ser 757, which disrupts AMPK-ULK1 interactions and suppresses autophagy (Kim et al., 2011). To nucleate autophagosomal membranes, the ULK complex phosphorylates components of the class III phosphatidylinositol 3-kinase (PI3K) complex (comprising VPS34, Beclin-1, ATG14 and VPS15), and generates phosphatidylinositol 3-phosphate (PI3P) on autophagosomal precursor membranes. Subsequently, PI3P facilitates the recruitment of the autophagy conjugation machinery, which includes ATG3, ATG7, and the ATG16L1-ATG5-ATG12 complex. Lipid conjugation of the ATG8 family members, including the microtubule-associated protein 1A/1B-light chain 3 (LC3) and GABARAP subfamilies, is mediated by these proteins, which play crucial roles in autophagosome maturation (Debnath et al., 2023; Zhao et al., 2021; Nakatogawa, 2020). A key step in autophagosome formation is the conjugation of the LC3/GABARAP proteins to the lipid phosphatidylethanolamine, and autophagy levels are frequently monitored by assessing these conjugation events (Miller and Thorburn, 2021).

### The critical roles of autophagy across distinct reproductive stages

Given the pivotal role of autophagy in diverse physiological processes, including stress responses and intracellular clearance, it has been postulated that autophagy is critically involved in the pathogenesis of human reproductive and associated disorders. Pregnancy establishment and maintenance involve several essential processes, including trophoblasts invasion, placentation, decidualization, enrichment and infiltration of decidual immune cells. Recent studies have suggested that autophagy contributes to the maintenance of pregnancy (Nakashima et al., 2019; Zhao X. et al., 2020). Here, the critical roles of autophagy throughout distinct stages of the reproductive process are discussed.

### Autophagy in gametogenesis and embryonic development

Gametogenesis is the process by which mature haploid gametes are formatted via meiosis and cell differentiation. In mice, autophagy activation occurs in the neonatal ovary, and contributes to mouse oogenesis (Moura et al., 2021). Knockout of the autophagy induction gene *Atg7* in germ cells results in subfertility in female mice, accompanied by severe loss of ovarian follicles (Song et al., 2015). Autophagy may prevent over-loss of oocytes by apoptosis in neonatal ovaries under starvation conditions. Further research has demonstrated that autophagy is essential for porcine oocyte maturation. During *in vitro* maturation of porcine oocytes, treatment with LY294002 (an autophagy inhibitor) impaired polar body extrusion, disrupted mitochondrial function, and triggered a DNA damage response and early apoptosis (Shen et al., 2018). *Atg14* knockdown does not affect mouse oocyte nuclear maturation, supporting the idea that autophagy’s role during oogenesis is species-dependent (Moura et al., 2021; You et al., 2016).

Autophagy also plays an important role in spermatogenesis. In *Atg7*-null spermatozoa, motility and morphological defects have been identified, linked to the disorganization of the flagella and other cytoskeletal components (Shang et al., 2016). Disruption of autophagy in male mice with Sertoli cell-specific knockout of *Atg5* or *Atg7*, results in disorganized seminiferous tubules and malformed spermatozoa (Liu et al., 2016). Conditional knockout of *Atg5* or *Atg7* in steroidogenic cells impairs autophagy, leading to reduced serum testosterone levels and abnormal sexual behavior in aging male mice (Gao et al., 2018).

Autophagy changes dynamically from fertilization to early embryonic development. It becomes activated during the 1-4 cell stage of the embryo to degrade excess maternal material in the fertilized egg (Zhao X. et al., 2020; Sato and Sato, 2013; Choi et al., 2013). In cross-fertilization experiments, using sperm from *Atg5*-null mice, oocytes from oocyte-specific *Atg5* knockout mice failed to progress beyond the four- or eight-cell stages due to autophagy defects (Tsukamoto et al., 2008). Inhibition of lysosomal function in mouse one-cell embryos, achieved through injection of short interfering RNAs targeting lysosome-associated membrane protein 1 and 2 (LAMP1 and LAMP2), led to developmental arrest at the two-cell stage (Tsukamoto et al., 2013). These findings confirm that autophagy is critical for early embryonic development.

### Autophagy in decidualization

Decidualization refers to the differentiation of endometrial stromal cells, ensuring the formation of a proper feto-maternal interface for regulated trophoblast invasion and correct placental orientation and growth (Mestre Citrinovitz et al., 2019). In humans, decidualization occurs independently of embryo implantation, and takes place during the luteal phase of each menstrual cycle, whereas in mice, it is triggered by blastocyst implantation (Ramathal et al., 2010).

Existing evidence suggests that autophagy induction is correlated with endometrial stromal cell decidualization. Autophagy is activated in decidualizing cells in both mice and humans (Mestre Citrinovitz et al., 2019; Rhee et al., 2016). The expression of *LC3-II* is higher in the decidua than in the proliferative or secretory phases of endometrial tissues (Su et al., 2020; Qin et al., 2022). Impaired uterine decidualization was observed when autophagy was inhibited by 3-methyladenine (3-MA) and chloroquine in an *in vivo* artificial decidualization mouse model. Conditional knockout of *Atg16L1* in the female reproductive tract reduces fertility by decreasing the implantation rate; endometrial stromal cells fail to properly decidualize, resulting in fewer implanted blastocysts. These findings in the absence of *Atg16L1* confirm the positive role of autophagy in the proper decidualization of endometrial stromal cells (Oestreich et al., 2020a). The depletion of FIP200, a key component of the ULK1 complex, impairs decidualization and endometrial receptivity in both mouse and human endometrial stromal cells (Oestreich et al., 2020b). In addition, studies have found that folate deficiency disrupts AMPK/mTOR signaling and autophagy, leading to abnormal endometrial decidualization and adverse pregnancy outcomes. These findings indicate that autophagy is essential for endometrial decidualization during early pregnancy mice (Zhang et al., 2021).

### Autophagy in placentation

Placentation establishes the interface between the fetus and mother, facilitating nutrients transport, gas exchange, waste excretion, and endocrine hormones secretion, critical processes for fetal development. It has been demonstrated that autophagy might be involved in placentation. The expression levels of *Atg5*, *Atg7* and *Atg16L1* continuously increase during the mouse placenta development (Qin et al., 2022; Chakraborty et al., 2020). Autophagy is predominantly induced in the decidua of rats, and inhibition of autophagy with 3-MA suppresses the differentiation of Rcho-1 cells into invasive trophoblasts, suggesting that autophagy is crucial for rat placentation (Arikawa et al., 2016). Enhanced autophagy has been observed in extravillous trophoblast (EVT) in early placental tissues. In autophagy-deficient EVT cells, invasion and vascular remodeling are significantly impaired under hypoxic conditions (Nakashima et al., 2013). Placenta-specific *ATG7* knockout female mice exhibit impaired placental growth and a significant increase in blood pressure, suggesting that placental autophagy is essential for normal placentation (Aoki et al., 2018).

However, studies investigating the relationship between autophagy and placentation have produced conflicting results. Folate deficiency *in vivo* leads to abnormalities in placental morphology, endocrine function, and expression of placental differentiation genes, accompanied by enhanced autophagy in the placentas. In addition, treatment with 3-MA inhibited placental autophagy and reversed placental impairment in mouse and human placental explants (Yin et al., 2019).

### Autophagy in maternal-fetal interface immune regulation

Studies have revealed that autophagy influences immune responses by regulating immune cell functions. Immune cells at the maternal-fetal interface play essential roles in pregnancy maintenance (Chen et al., 2023). An inappropriate immune response is often associated with pregnancy failure. During pregnancy, the human decidua contains numerous immune cells, including innate immune cells, such as natural killer (NK) cells, macrophages, and dendritic cells (DCs), as well as adaptive immune cells such as CD8^+^ T cells, CD4^+^ T cells and regulatory T cells (Tregs) (True et al., 2022; Mor, 2022). Different stages of pregnancy require unique immunological environments to provide support and protection. Implantation and early placentation depend on an inflammatory response, whereas fetal growth is characterized by immune tolerance. Ultimately, a return to an inflammatory environment is necessary during parturition (Mor et al., 2017). The transition between a pro- and an anti-inflammatory state is essential for pregnancy maintenance.

### NK cells

Autophagy is the primary regulator of both innate and adaptive immunity. The absence of *Atg5* leads to progressive mitochondrial damage, reactive oxygen species (ROS) accumulation, and regulated cell death in NK cells, thereby interfering with their development and function (López-Soto et al., 2017). In a co-culture system, stimulating autophagy with rapamycin in human trophoblast cells significantly reduced NK cell cytotoxicity. Conversely, administrating 3-MA in a pregnant mouse model enhanced uterine NK cells cytotoxicity, and increased the embryo absorption rate (Tan et al., 2020). Increased autophagy in decidual stromal cells facilitates the adhesion and retention of decidual NK cells by activating the MITF-TNFRSF14/HVEM signaling pathway during normal pregnancy (Lu et al., 2021). Overall, autophagy may play a role in the regulating of NK cell function at the maternal-fetal interface.

### Macrophages

Macrophages are the second-largest population of immune cells in the decidua, primarily responsible for regulating immune tolerance and protecting against infections. Insufficient autophagy in decidual macrophages impairs their function and increases the risk of spontaneous abortion (Yang H. L. et al., 2022). Autophagy is essential for macrophage differentiation and polarization. During monocyte undergo differentiation, autophagy is induced, and its inhibition leads to the apoptosis of the differentiated cells (Zhang et al., 2012). Therefore, autophagy is pivotal for monocyte survival and differentiation. Kupffer cells and bone marrow-derived macrophages from *Atg5* knockout mice display abnormal polarization, characterized by proinflammatory M1 and decreased anti-inflammatory M2 polarization (Liu et al., 2015). In *Atg7* knockout mice, monocytes exhibit impaired differentiation into M2 macrophages, accompanied by increased glycolytic activity and inflammatory cytokine production (Chen et al., 2023; Stranks et al., 2015). These findings suggest that autophagy plays an important role in macrophage polarization.

### Dendritic cells

Despite representing only a small proportion of leukocytes in decidua, DCs play a critical role in balancing immune responses and maintaining tolerance. They closely interact with other immune cells such as T cells, NK cells and macrophages (Mori et al., 2016; Wei et al., 2021). In addition, DCs are among the most efficient antigen presenting cells and play a critical role in activating naïve T cells, thereby promoting protective immunity against infections and maintaining immune tolerance (Ghislat and Lawrence, 2018). Autophagy is involved in the tolerogenic and immunogenic functions of DCs. Cannabinoid induced autophagy promotes the generation of human tolerogenic DCs, which polarize functional FOXP3^+^ Tregs (Angelina et al., 2022). The synthetic cannabinoid WIN55212-2 exerts anti-inflammatory effects in lipopolysaccharide (LPS)-induced sepsis through CB1- and PPARα-mediated autophagy induction and promotes the generation of FOXP3^+^ Tregs. *Atg16L1* deficiency induced DC hyperactivity is associated with an increase expression of Laptm5, a proinflammatory lysosomal protein that enhances NF-κB signaling by inhibiting the ubiquitin-editing enzyme A20 (Hubbard-Lucey et al., 2014). *Atg7* deficiency in DCs significantly reduces the onset and severity of experimental autoimmune encephalomyelitis, due to reduced T cell priming (Bhattacharya et al., 2014). Similarly, mice with DC-specific *Atg5* deletion exhibit a reduced CD4^+^ T-cell priming, while CD8^+^ cytotoxic T-cell priming remains unaffected (Oh and Lee, 2019). However, *Atg5*-deficient DCs exhibit an enhanced CD8A^+^ T-cell response and increased secretion of proinflammatory cytokines following respiratory syncytial virus (RSV) infection, accompanied by elevated glycolytic activity and activation of the *AKT*-*mTOR*-*RPS6KB1* signaling pathway (Oh et al., 2021). Collectively, these findings suggest that autophagy is essential for proper DC function.

### Autophagy in pregnancy complications

**FIGURE 2:**
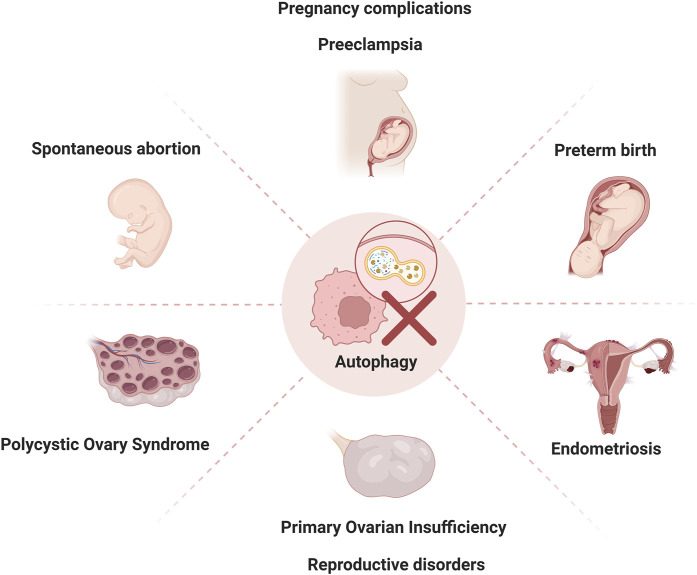
Autophagy dysregulation in reproductive disorders Autophagy dysregulation contributes to pathogenesis of reproductive disorders, including spontaneous abortion, preeclampsia, preterm birth, polycystic ovary syndrome, premature ovarian insufficiency, and endometriosis.

While autophagy plays a vital role in maintaining cellular homeostasis during normal pregnancy, its dysregulation has been increasingly implicated in various pregnancy complications (Figure 2). Understanding these aberrant autophagic mechanisms not only sheds light on disease pathogenesis but also opens avenues for potential therapeutic interventions. The following section explores how autophagy contributes to specific gestational disorders, bridging the gap between fundamental mechanisms and clinical implications.

### Autophagy and spontaneous abortion

Spontaneous abortion (SA) occurs in approximately 10%–15% of pregnancies (Lu et al., 2021). Its etiology is multifactorial, involving chromosomal abnormalities, uterine anatomical defects, endocrine disorders, endometrial infections, and immune factors (Dimitriadis et al., 2020). However, the underlying causes of SA remain unclear in a significant proportion of SA cases. Abnormal autophagy levels are associated with the onset of SA. Patients with unexplained SA display insufficient autophagy in decidual stromal cell and resistance of decidual natural killer (dNK) cell. In addition, low doses of rapamycin significantly promote endometrial autophagy and NK cell residence, improving embryo implantation in spontaneous abortion mouse models (Lu et al., 2021). There is a significant decrease in autophagy in the villi of recurrent spontaneous abortion (RSA) patients compared to patients undergoing elective pregnancy termination, resulting in impaired trophoblast cell invasion (Tan et al., 2020). The expression of *PVT1* in RSA villus is significantly reduced. *PVT1* knockdown significantly reduced autophagy and invasion in HTR-8/SVneo cells (Yang et al., 2020). However, the role of autophagy in the development of SA remains unclear. Recent studies have reported that the expression of autophagy related proteins is markedly upregulated in decidual macrophages of RSA patients (Yang Y. et al., 2022). Upregulation of *HMGB1* in villous tissues and a high level of placental autophagy have been observed in patients with early unexplained miscarriage. In addition, inhibition of *HMGB1* and autophagy reversed the proliferation and migration of LPS-induced HTR-8/SVneo cells (Zhou F. et al., 2021).

Pan et al. have also demonstrated that Shh signaling is significantly impaired in human placental tissue from patients with RSA compared to normal controls. Inhibition of Shh signaling triggers autophagy and leads to autolysosome accumulation (Pan et al., 2021). These inconsistent results across studies may be due to differences in cell types and methodologies used to evaluate autophagy. Collectively, these studies suggest that dysregulated autophagy is associated with SA.

### Autophagy and preeclampsia

Clinical manifestations of preeclampsia (PE) include new-onset hypertension and proteinuria after 20 weeks of gestation in previously normotensive women. PE is a serious pregnancy complication, and its etiology remains largely unknown. Multiple contributing factors have been identified, including uteroplacental ischemia, maternal infection and inflammation, gut dysbiosis, obesity, fetal abnormalities, autoimmune diseases, placental aging, disrupted maternal-fetal immune tolerance, and endocrine disorders (Jung et al., 2022). Autophagy has been involved in the pathophysiology of PE. *Atg7* conditional knockout mice exhibit poor trophoblast invasion, increased blood pressure, decreased placental growth factor levels, and small placentas during pregnancy, which are key features of preeclampsia placentas (Aoki et al., 2018; Sharma, 2018). Akitoshi et al. provided evidence of defective autophagy in placental tissues from patients with PE. Autophagy suppression promotes the accumulation of toxic protein aggregates, which may impair placentation and create a pregnancy-incompatible milieu (Nakashima et al., 2020). Cyclosporin A reduces placental necrosis and senescence by upregulating autophagy, and improving symptoms in PE-like mice (Hu et al., 2022). However, there are some conflicting data. Gu et al. reported enhanced autophagy in placental tissue from human PE patients, as well as in, PE mouse model, and cellular model. Esomeprazole treatment inhibits placental autophagy and alleviates PE symptoms by inhibiting *AMPKα* and activating mTOR signaling (Gu et al., 2022). Suppression of PKCβ has been observed in PE, and blocking PKCβ in pregnant mice leads to autophagy activation and induces PE like phenotypes such as fetal growth restriction, proteinuria, and hypertension (Zhao H. et al., 2020). In summary, autophagy abnormalities are associated with the development of PE, however, further more rigorous experimental studies are needed.

### Autophagy and preterm birth

Preterm birth is the leading cause of perinatal mortality. The etiological factors and pathogenic mechanisms of preterm birth encompass genetic and epigenetic predispositions, maternal and fetal stress with CRH pathway dysfunction, inflammatory and infectious processes, and progesterone-related hormonal imbalance (Pisacreta and Mannella, 2022). Autophagy has been implicated in the pathological mechanism underlying preterm birth. Single nucleotide polymorphisms (SNPs) in the *ATG16L1* gene lead to reduced autophagy activity, and this polymorphism is associated with a faster progression from induction to delivery in women with an unfavorable cervix (Doulaveris et al., 2013). The placentas of women with preterm birth indicates decreased autophagic activity, which is associated with elevated levels of infection markers. In mice, reduced *ATG16L1* expression results in preterm birth upon an inflammatory stimulation and increases susceptibility to infection in *ATG16L1*-deficient placentas (Cao et al., 2016). Increased mTORC1 and COX2 signaling has been observed in women with preterm births. In addition, inhibition of mTORC1 signaling by rapamycin rescues preterm birth (Cha et al., 2013; Hirota et al., 2011). The expression of autophagy related genes *Atg4c* and *Atg7* is significantly decreased in the mouse placenta and uterus during inflammation-induced preterm birth, whereas no such decrease is observed during hormonally induced preterm birth. Altered autophagy activates the NF-κB p65 pathway, amplifying the inflammatory response and leading to preterm birth (Agrawal et al., 2015). These studies suggest that changes in autophagy are closely related to preterm birth, and that targeting autophagy may offer therapeutic potential for its treatment.

### Autophagy in reproductive disorders

While dysregulated autophagy has been established as a pivotal factor in pregnancy complications such as spontaneous abortion and preeclampsia, its pathological significance extends beyond the gestational period. Emerging evidence indicates that autophagy also plays critical roles in reproductive disorders. For instance, impaired folliculogenesis in polycystic ovary syndrome (PCOS), accelerated follicle depletion in primary ovarian insufficiency (POI), and lesion survival in endometriosis are all mechanistically linked to aberrant autophagic activation or suppression.

### Autophagy and PCOS

PCOS is a common endocrine and metabolic disorder syndrome affecting women of reproductive age that leads to reproductive dysfunction. PCOS is a heterogeneous disorder characterized by a combination of various signs and symptoms, including androgen excess, polycystic ovarian morphology, and ovulatory dysfunction (manifested as oligo-ovulation or anovulation) (Helvaci and Yildiz, 2025). The reproductive-related implications of PCOS encompass irregular menstrual cycles, anovulatory infertility, increased risks of pregnancy complications, and endometrial cancer, among other aspects (Stener-Victorin et al., 2024).

Recent studies have advanced our understanding of the role of autophagy in the pathogenesis of PCOS. Autophagy is significantly enhanced in the ovarian tissues of both humans, rats and mice with PCOS. Specifically, the autophagy marker protein *LC3B* is elevated in the ovarian granulosa cells of PCOS rats. Compared with normal ovarian tissue, the ratio of LC3-II to LC3-I is markedly increased in the ovarian tissue from patients with PCOS (Li et al., 2018). In PCOS patients with comorbid insulin resistance (IR), High mobility group box 1 (HMGB1) levels in follicular fluid are significantly elevated, accompanied by enhanced autophagy in granulosa cells. HMGB1 activated granulosa cell autophagy by increasing the LC3B II/I ratio and ATG7 expression while reducing p62 levels. Blocking the autophagy pathway reverses HMGB1-induced insulin resistance effects, suggesting that elevated HMGB1 levels promote the development of insulin resistance in granulosa cells of PCOS patients through exacerbated autophagy (Zhang et al., 2020).

The mechanisms underlying the excessive activation of autophagy in ovarian tissue of patients with PCOS remain unclear. Studies in mice have revealed that dihydrotestosterone (DHT) significantly upregulates Wnt5a protein levels in granulosa cells. Downregulation of Wnt5a effectively suppresses autophagy in PCOS granulosa cells by activating the PI3K/AKT/mTOR signaling pathway, thereby ameliorating ovarian dysfunction and hyperandrogenism in a PCOS mouse model (Ma et al., 2025). In PCOS patients, Block of Proliferation 1 (BOP1) mRNA levels are negatively correlated with antral follicle count (AFC), body mass index (BMI), and serum androgen levels. Mechanistically, BOP1 knockdown triggered the nucleolar stress response, promoting the release of RPL11 from the nucleolus to the nucleoplasm. This inhibits the E3 ubiquitin ligase activity of MDM2, enhancing the stability of p53 protein. Subsequently, TP53 suppresses the mTOR signaling pathway, which activates autophagy in granulosa cells. Local ovarian injection of a lentiviral vector overexpressing BOP1 significantly inhibits autophagy and ameliorates hyperandrogenism, estrous cycle irregularities, and abnormal follicular development in a PCOS mouse mode (Ji et al., 2024). Following androgen stimulation, Ferredoxin 1 (FDX1) expression was upregulated in granulosa cells. FDX1 regulates autophagy by modulating the autophagy-related proteins ATG3 and ATG7. This study confirms that FDX1 plays a critical role in female folliculogenesis by mediating autophagy (Xing et al., 2023). Despite extensive research, the role of autophagy in PCOS pathogenesis and the regulatory mechanisms underlying aberrant autophagy in PCOS remain to be systematically and thoroughly investigated.

### Autophagy and POI

POI, also known as premature ovarian failure (POF), is characterized by an abnormal decline of ovarian function before the age of 40 in women (Federici et al., 2024). POI primarily manifests as menstrual disorders, elevated gonadotropin levels (follicle-stimulating hormone [FSH] >25 U/L), and fluctuating decreases in estrogen levels. Patients with POI often develop various complication, including infertility, Alzheimer’s disease, osteoporosis, and cardiovascular diseases (Verrilli, 2023). POI exhibits high etiological heterogeneity encompassing genetic, autoimmune, iatrogenic, and infectious factors, while its exact pathogenesis remains incompletely understood.

Numerous studies have confirmed the correlation between dysregulation of autophagy and the onset of POI. Disrupted autophagy can lead to defective germ cell survival, resulting in increased apoptosis and follicle atresia (Ding et al., 2024). Germ cell-specific knockout of *ATG7* induces autophagy disruption, leading to reproductive defects with severe follicular depletion in female mice, presenting POI-like phenotypes (Song et al., 2015). Studies have identified mutations in autophagy-related genes, specifically the *ATG7 p. Phe403Leu* and *ATG9A p. Arg758Cys* variants, in patients with POI. Functional investigations demonstrated that these genetic variants significantly impair cellular autophagy, indicating that autophagy represents a novel pathophysiological mechanism underlying human POI (Delcour et al., 2019). *EPG5* knockout blocks autophagic flux and induces POI-like phenotypes in female mice. Mechanistically, *EPG5* deficiency significantly upregulates the transcription factor WT1 at the protein level, which subsequently represses the expression of steroidogenic genes in granulosa cells of antral follicles (Liu et al., 2023). A pathogenic variant of *CKAP5* has been identified in patients with POI, resulting in protein truncation and loss of function. *Ckap5* heterozygous knockout mice recapitulated the POI phenotype, which is characterized by a reduced primordial follicle reserve and accelerated follicular atresia. *CKAP5* deficiency impairs ovarian DNA damage repair and autophagy via *ATM* and *ATG7*, ultimately leading to increased follicular apoptosis, reduced oocyte quantity, and impaired oocyte quality (Hu et al., 2025). *Tet1*-deficient mice exhibit significantly diminished ovarian follicle reserves at a young age, which progressively declines with age, phenocopying POF. Single-cell transcriptomic analysis of oocytes revealed that *Tet1* deficiency is associated with impaired ubiquitination and defective autophagy (Liu et al., 2021). Resveratrol treatment upregulates IL-6 levels in the ovaries and ameliorates POI progression in mice. IL-6 activates granulosa cells via soluble IL-6 receptor (sIL-6R), thereby promoting autophagy in granulosa cells. Resveratrol and IL-6 synergistically enhance autophagy in granulosa cells (Hu et al., 2024).

Multiple molecular mechanisms contribute to the autophagy dysregulation in POI pathogenesis. Impaired DNA repair triggers excessive autophagy, leading to autophagic cell death and germ cell depletion. Epigenetic modifications, including DNA methylation and demethylation, regulate autophagy-related genes, and abnormalities in these processes can disrupt autophagic function. Additionally, oxidative stress induced by various factors exacerbates autophagy impairment (Ding et al., 2024).

### Autophagy and endometriosis

Endometriosis is characterized by the ectopic growth of endometrial tissue outside the uterine cavity (primarily on the ovaries and pelvic peritoneum), affecting approximately 10% of women of reproductive age worldwide (Shen et al., 2021). Clinically, it presents with chronic pelvic pain, dysmenorrhea, and infertility. The pathogenesis of endometriosis involves multiple factors, including cellular adhesion and proliferation, local inflammation, ectopic steroidogenesis, neurogenesis, and immune dysregulation (Allaire et al., 2023). Although various theories have been proposed, the underlying mechanisms remains incompletely understood.

Aberrant autophagy in both the eutopic endometrium and ectopic endometriotic lesions contributes to disease progression by promoting the hyperplasia of ectopic tissues and stromal cells, suppressing apoptosis, and inducing abnormal immune responses. The study revealed significantly upregulated expression of autophagy-related markers *BECN-1*, *Atg13* and *SQSTM1* in the ectopic endometrium of patients with endometriosis (Huang et al., 2021). Macrophages in the peritoneal fluid of affected patients exhibit significantly reduced expression of hematopoietic cellular kinase (*HCK*). This deficiency further upregulates macrophage autophagy in a c-FOS/c-JUN-dependent manner, resulting in impaired macrophage phagocytic function. Pretreatment with the autophagy inhibitor Bafilomycin A1 restores macrophage phagocytic function and suppresses endometriosis progression (Lei et al., 2024). The Indian hedgehog signaling pathway is significantly suppressed in the endometrial tissues of patients with endometriosis, which subsequently activates endometrial cell autophagy and promotes the abnormal survival of ectopic endometrial cells (Zhou Y. et al., 2021). However, research findings regarding the role and underlying mechanisms of autophagy in endometriosis remain inconsistent. Some studies suggest that under conditions of high estrogen concentrations and progesterone resistance, alterations occur in autophagy-related genes, leading to decreased autophagic activity in the endometrium. This suppression of autophagy directly accelerates the implantation, growth, and angiogenesis of endometriotic lesions (Shen et al., 2021). These inconsistent findings require further in-depth and extensive validation.

Multiple factors contribute to the development of aberrant autophagy in endometriosis, involving pathophysiological processes such as female hormones, hypoxia, and oxidative stress. Endometriosis is characterized by estrogen dependence and progesterone resistance, with high estrogen levels and progesterone resistance g considered key regulatory factors leading to abnormal autophagy in patients (Shen et al., 2021; Yang et al., 2017). Hypoxia-mediated upregulation of *ADAR1* suppresses circFOXO3 expression, and the consequent loss of circFOXO3 induces autophagy by impairing p53 degradation, thereby contributing to the pathogenesis of endometriosis (Zhang et al., 2024). Deficient MST1 expression has been observed in the peritoneal macrophages of patients with endometriosis. *MST1*-deficient macrophages secrete the anti-inflammatory cytokine IL-10, which promotes autophagy in ectopic endometrial stromal cells (Huang et al., 2022).

## Conclusion

Numerous studies have definitively confirmed the close association between autophagy dysregulation and reproductive disorders, and this review synthesizes the latest advances in this field. This review systematically elucidates the dual regulatory role of autophagy in reproductive physiology and pathology. Currently, the mechanistic role of autophagy dysregulation in disease pathogenesis remains debated, necessitating more rigorous and precise methodologies to assess autophagic activity in humans. Future therapeutic strategies targeting autophagy modulation show promise for managing reproductive disorders, though their clinical implementation remains contingent upon more rigorous research validation.

## Abbreviations

CMA, chaperone-mediated autophagy; ATG, autophagy-related genes; ULK, UNC-51-like kinase; mTORC1, mechanistic target of rapamycin complex 1; PI3P, phosphatidylinositol 3-phosphate; LC3, microtubule-associated protein 1A/1B-light chain 3; LAMP, lysosome-associated membrane protein; 3-MA, 3-methyladenine; EVT, extravillous trophoblast; NK, natural killer cells; DCs, dendritic cells; Tregs, regulatory T cells; SA, spontaneous abortion; PE, Preeclampsia; PCOS, Polycystic ovary syndrome; POI, Primary ovarian insufficiency.
